# Differentiation in Nitrogen Transformations and Crop Yield as Affected by Tillage Modes in a Fluvo-Aquic Soil

**DOI:** 10.3390/plants12040783

**Published:** 2023-02-09

**Authors:** Fengmin Shen, Changwei Zhu, Guiying Jiang, Jin Yang, Xuanlin Zhu, Shiji Wang, Renzhuo Wang, Fang Liu, Xiaolei Jie, Shiliang Liu

**Affiliations:** College of Resources and Environmental Sciences, Henan Agricultural University, Zhengzhou 450002, China

**Keywords:** rotation tillage, fluvo-aquic soil, wheat-maize system, nitrogen forms, nitrogen distribution

## Abstract

Nitrogen is a vital element for soil fertility and crop productivity. The transformation of nitrogen is directly affected by tillage practices for the disturbing soil. The characteristics of different nitrogen forms under different tillage modes are still unclear. A 3-year cycle tillage experiment was carried out to assess the combination of rotary tillage (RT), deep tillage (DT), and shallow rotary tillage (SRT) on nitrogen transformation and distribution, wheat yield and nitrogen balance in fluvo-aquic soil from Huang-Huai-Hai Plain in China. The results showed the rotation tillage cycle with deep tillage in the first year increased the total nitrogen (TN), and the main nitrogen form content in 0–30 cm compared with continued rotary tillage (RT-RT-RT). Moreover, the nitrate (NO_3_^−^-N) and ammonium nitrogen (NH_4_^+^-N) content were improved in 20–40 cm by deep tillage practice with the highest value as 39.88 mg kg^−1^ under DT-SRT-RT. The time, tillage, and depth significantly affected the different nitrogen forms, but there was no effect on dissolved organic carbon (DON) and soil microbial biomass nitrogen (SMBN) by the interaction of time and tillage. Moreover, compared with RT-RT-RT, the rotation tillage promoted the spike number and kernels per spike of wheat, further increasing the wheat yield and nitrogen partial productivity, and with a better effect under DT-SRT-RT. The NO_3_^−^-N and NH_4_^+^-N trended closer and positively correlated with wheat yield in 0–40 cm in 2019. The rotation tillage with deep tillage improved the different forms of nitrogen in 0–30 cm, wheat yield, and nitrogen partial productivity, and decreased the apparent nitrogen loss. It was suggested as the efficiency tillage practice to improve nitrogen use efficiency and crop yield in this area.

## 1. Introduction

Nitrogen is the necessary element for the plant, which directly decides the crop yield [1]. The nitrogen transformation is affected by various factors, such as tillage, irrigation, fertilization, and so on. Tillage practice is a common agricultural practice to directly disturb and change the soil’s physical properties, further affecting soil nutrient conversion and crop productivity [2]. The disturbance degree on soil varies from different tillage methods. Therefore, the effect of different tillage practices on soil physicochemical properties from different depths is different. Moreover, the tillage not only affects the change in soil nitrogen content, but also affects the profile distribution of soil nitrogen due to the downward shift of nitrogen and the effect of crop roots and may therefore influence crop yield and quality [3,4].

Soil nitrogen exists as organic components, its transformation in the soil is essentially associated with the interconversion of inorganic forms such as ammonium (NH_4_^+^-N), nitrate (NO_3_^−^-N), and organic components [5], and is regulated by interactive processes of production and consumption [6]. The transformation process is driven by soil microorganisms. Soil microbial biomass nitrogen (SMBN) and dissolved organic nitrogen (DON) are important labile soil organic nitrogen (SON) fractions, which are considered actively involved in N mineralization and are potentially more sensitive indicators for agricultural management [7,8]. Although DON only accounts for 0.15–0.19% of soil TN, it is one of the relatively active components of the soil organic nitrogen pool and has important effects on nitrogen transformation and the environment. DON also represents a source of energy and nutrients for microbial growth and activity [9]. Tillage practice directly affects this process by changing the soil microenvironment, such as soil structure, and regulating the soil temperature and moisture, further mediating soil microorganism community and structure, and finally determining the nitrogen transformation. Studies have shown that conservation tillage usually increased soil N content compared to conventional tillage [2,3,10,11,12], but long-term no-till and reduced tillage produce nutrient accumulation and N mineralization in the soil surface [13], and may also cause topsoil compaction [14], leading to a reduction in the air-filled pore space [15,16], which may decrease the root absorption of soil nutrients and water [17]. Minimum tillage, often in combination with other practices, has been promoted to improve soil health through enhanced microbial activity and increased soil organic matter (SOM) in the surface layer [18,19,20]. long-term no-till management is a benefit for soil N stocks, N mineralization, and efficient fertilizer N use in corn-based cropping systems on well-drained soils. A meta-analysis by Mahal et al. [21] found that no-till increased potentially mineralizable N relative to moldboard plowing. Tillage can increase rates of soil C and N mineralization by disrupting aggregates and incorporating residue. Yuan et al. [6] found that no-tillage combined with maize straw mulching could simultaneously maintain the retention and availability of soil N to achieve effective N cycling in agroecosystems. Meanwhile, nutrients concentrated near the surface of no-till soils may increase the possibility of loss via erosion, runoff, and volatilization. Nutrient stratification may also reduce the availability and uptake of nutrients by crops.

In contrast, deep tillage can loosen the soil and promote crop roots grow and absorb more soil nutrients, but it may increase the loss of soil nitrogen and other nutrients, and accelerate soil erosion, cause serious environmental pollution and soil degradation thereby affecting ecosystem functions in the field [22,23]. Therefore, rotational tillage with the combination of different tillage practices was suggested to dismiss the disadvantage of long-term mono-conventional tillage [24]. Rotation tillage techniques that reasonably combined different tillage measures can effectively counteract some defects caused by mono-tillage practices [25,26,27]. In recent years, periodic disturbance of continuous NT systems in the form of occasional tillage, one-time tillage, strategic tillage, targeted tillage, single inversion tillage, one cycle of tillage, etc., has been promoted as a potential strategy to address the challenges of long-term NT management [28,29,30,31].

Huang-Huai-Hai Plain is the main agricultural region in China, with mainly wheat and maize double cropping systems. The long-term mono-rotary tillage with soil disturbance depth of around 15 cm in this area leads to a shallow plow layer and thick plow bottom, leading to the uncoordinated supply of soil water, fertilizer, gas, and heat, restricts the extension of crop roots, and impedes the increase in crop yields. Thus, optimum tillage practices are essential for crop production in this area. Therefore, the objectives of this study are (ⅰ) to clarify the differentiation in nitrogen transformation and distribution by the different tillage modes (ⅱ) to assess the effect of different tillage modes on crop yield, nitrogen use efficiency, and nitrogen balance (ⅲ) to select the optimum tillage mode according to the above results.

## 2. Results

### 2.1. Distribution of Soil Total Nitrogen under Different Tillage Modes

**Figure 1:**
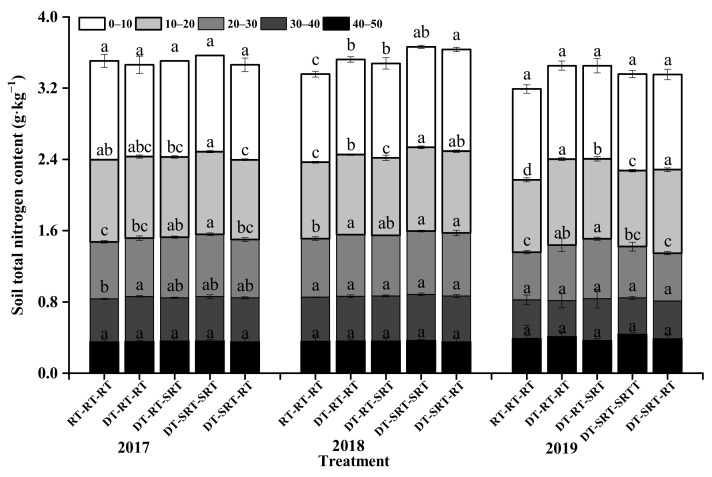
Soil total nitrogen content in different soil layers under different treatments. Note: Different lowercase letters indicate significant differences between different treatments at the same soil layer (*p* ≤ 0.05).

The total nitrogen content (TN) decreased with soil depth under all tillage treatments during 2017–2019 (Figure 1). The tillage affects the TN in the 0–40 cm soil layer in 2017 and the effect decreased within 0–30 cm in 2018 and 2019. TN content was no different in 0–10 cm and 40–50 cm in 2017 among the treatments, while it was significantly higher under the treatments with deep tillage combination in 20–40 cm than that under RT-RT-RT. During 2018 and 2019, the deep tillage increased TN content in 0–30 cm soil layer compared with RT-RT-RT, with the higher one under DT-SRT-SRT and DT-SRT-RT as 1.14 g kg^−1^ and 1.13 g kg^−1^, respectively, in 2018. 

### 2.2. Distribution of Soil Alkaline Nitrogen under Different Tillage Modes

**Figure 2:**
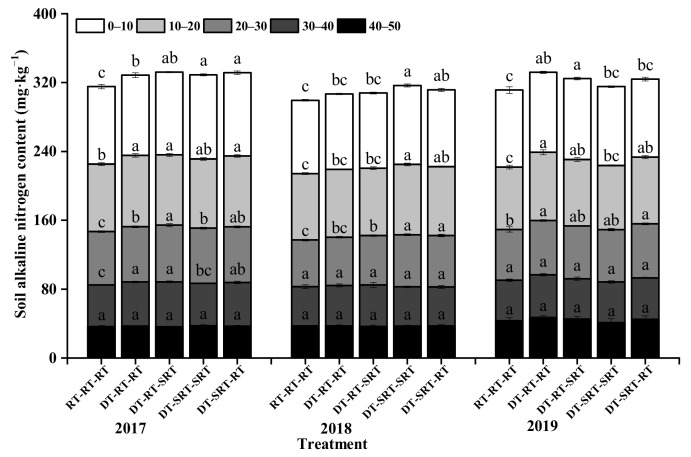
Soil alkaline nitrogen content in different soil layers under different treatments. Note: Different lowercase letters indicate significant differences between different treatments at the same soil layer (*p* ≤ 0.05).

Similar to TN, the alkaline nitrogen content (AN) under all treatments was decreased with the increase in soil depth during 2017–2019 (Figure 2). Compared with RT-RT-RT, the AN was affected by treatments with deep tillage in 0–40 cm in the first year (2017), and the effect decreased within 0–30 cm during the following two years. During the three-year experiment, the AN content was increased under the treatments with deep tillage compared with that under RT-RT-RT. While among the treatments with deep tillage, AN content did not demonstrate a significantly different or clear trend. It indicated that the effect of deep tillage was bigger than the combined other two tillage modes.

### 2.3. Distribution of Soil Nitrate Nitrogen under Different Tillage Modes

**Figure 3:**
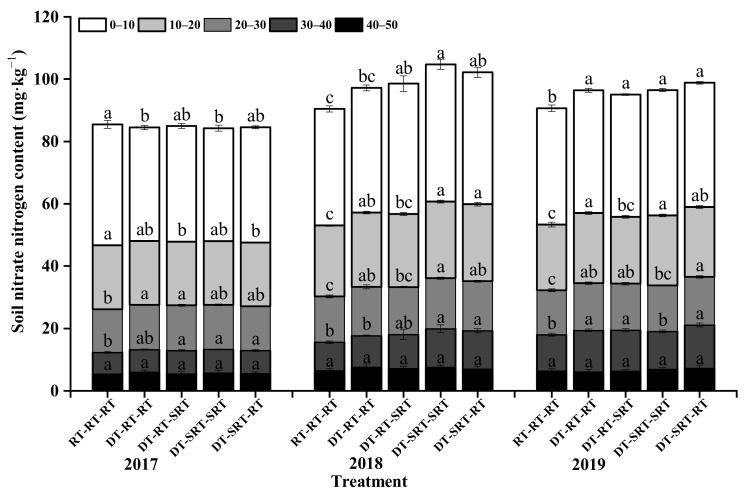
Soil nitrate nitrogen content in different soil layers under different treatments. Note: Different lowercase letters indicate significant differences between different treatments at the same soil layer (*p* ≤ 0.05).

The nitrate nitrogen (NO_3_^−^-N) content under all treatments was decreased with the increase in the soil depths, and the effect of tillage on NO_3_^−^-N in 0–40 cm during 2017–2019 (Figure 3). In the first year, the NO_3_^−^-N content in 0–20 cm soil layer under RT-RT-RT did not differ from that under the treatments with deep tillage, while, which was lower under RT-RT-RT in 0–40 cm during the following two years. Meanwhile, the NO_3_^−^-N content in 30–40 cm soil layer under treatment with deep tillage increased with time, the NO_3_^−^-N content under DT-SRT-SRT treatments was significantly higher than that under RT-RT-RT treatments, the highest increase was 35.12% in 2018. In 2019, the NO_3_^−^-N content in the 0–40 cm soil layer was significantly increased under DT-SRT-RT with the highest value of 39.88 mg kg^−1^. This indicated that the deep tillage accelerated the NO_3_^−^-N leaching into the deeper soil layer.

### 2.4. Distribution of Soil Ammonium Nitrogen under Different Tillage Modes

**Figure 4:**
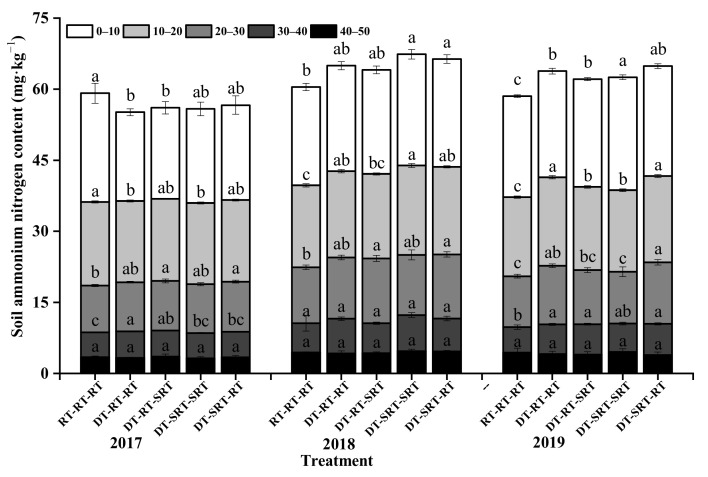
Soil ammonium nitrogen content in different soil layers under different treatments. Note: Different lowercase letters indicate significant differences between different treatments at the same soil layer (*p* ≤ 0.05).

The ammonium nitrogen (NH_4_^+^-N) content under all treatments decreased with the increasing soil depths, and it slightly increased with time. The effect of tillage on NH_4_^+^-N in 0–40 cm during 2017–2019 (Figure 4). Similar to NO_3_^−^-N, the NH_4_^+^-N content in 0–20 cm soil layer under RT-RT-RT did not differ from that under the treatments with deep tillage, while it was lower under RT-RT-RT in 0–40 cm during the following two years. Meanwhile, the NH_4_^+^-N content in 0–30 cm soil layer under treatment with deep tillage significantly increased with time, the NH_4_^+^-N content under DT-SRT-SRT treatments was significantly higher than that under RT-RT-RT treatments, the highest increase was 35.12% in 2018. In 2019, the NH_4_^+^-N content in the 0–40 cm soil layer was significantly higher under DT-SRT-SRT than that under RT-RT-RT with the highest value of 23.82 mg kg^−1^ in the 0–10 cm soil layer.

### 2.5. Distribution of Soil Dissolved Organic Nitrogen under Different Tillage Modes

**Figure 5:**
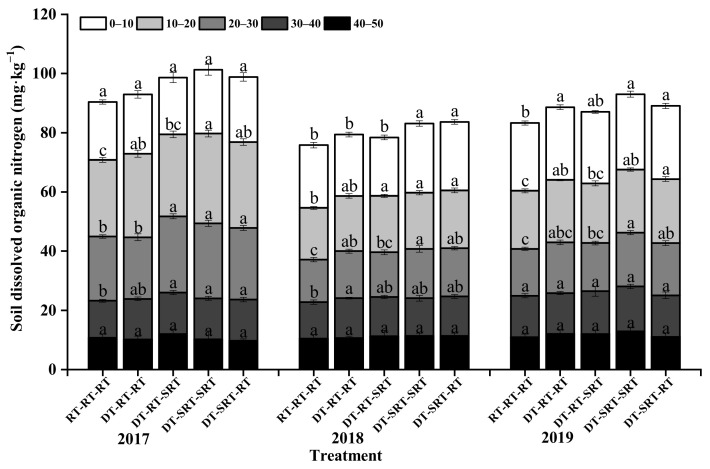
Soil dissolved organic nitrogen content in different soil layers under different treatments. Note: Different lowercase letters indicate significant differences between different treatments at the same soil layer (*p* ≤ 0.05).

The soil dissolved organic nitrogen content (DON) under all treatments decreased first and then slightly increased with time. The major change happened in the 10–30 cm soil layer (Figure 5). The DOC content did not differ from all treatments in 0–10 cm in 2017, but it decreased under RT-RT-RT in the following two years. The DOC was no different in the 40–50 cm soil layer in 2017 and 2018, while it was in the 30–50 cm soil layer in 2019. It indicated that the effect of deep tillage decreased in the third year. Although the DON, generally, was no different among the treatments with deep tillage, the DON under DT-SRT-SRT and DT-SRT-RT was significantly higher than that under RT-RT-RT in 0–30 cm in 2018 and 2019, with the highest value of 23.37 and 25.49 mg kg^−1^ under DT-SRT-SRT in the 0–10 cm soil layer.

### 2.6. Distribution of Soil Microbial Biomass Nitrogen under Different Tillage Modes

**Figure 6:**
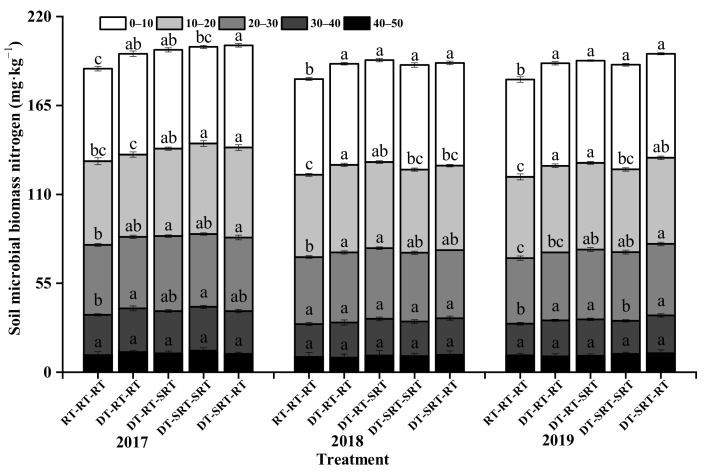
Soil microbial biomass nitrogen content in different soil layers under different treatments. Note: Different lowercase letters indicate significant differences between different treatments at the same soil layer (*p* ≤ 0.05).

The microbial biomass nitrogen content (SMBN) under all treatments decreased with soil depth increasing during 2017–2019 (Figure 6). Although the SMBN slightly fluctuated during the three years, it increased under the treatments with deep tillage compared with DT-SRT-RT in the 0–40 cm soil layer in 2017 and 2019, and in 0–30 cm in 2018. Generally, SMBN did not differ from the treatments with deep tillage in all the depths and years. The SMBN was significantly higher under DT-RT-SRT than under RT-RT-RT in the three-year experiment, with the highest values of 61.06, 63.03, and 63.26 mg kg^−1^ in 0–10 cm in the three years.

### 2.7. The Three-Factor Analysis with Time, Tillage, and Soil Depth on Nitrogen Forms

The multivariate analysis demonstrated that all nitrogen forms were affected by tillage time (year), tillage modes, and soil sample depth, respectively (Table 1). Therein, the effect of soil depth was the most important factor in the different nitrogen forms. All the nitrogen forms were affected by the interaction of tillage time and sample depth, tillage mode, and sample depth. However, there was no interaction effect on DOC and SMBN by tillage time and tillage mode.

### 2.8. Wheat Yield, Yield Component, and Fertilizer Partial Productivity

The wheat yield, yield components, and nitrogen partial productivity changed over different years (Table 2). In the first year (2017), the spike number and thousand kernel weight did not differ from treatments, the difference in yield was driven by kernels per spike. The higher wheat yield was found under RT-RT-RT and DT-RT-RT with 6717 and 6383 kg ha^−1^, respectively. In 2018 and 2019, generally, the wheat yield and yield component all showed higher under the treatments with deep tillage compared with RT-RT-RT. The highest wheat yield was under DT-SRT-RT in 2018 and 2019 with 6346 and 6557 kg ha^−1^, respectively. The N partial productivity demonstrated a similar trend with wheat yield, with a higher value of 28.98 and 29.94 kg kg^−1^ in 2018 and 2019, respectively.

### 2.9. The Nitrogen Balance under Different Treatments

The nitrogen balance was calculated in 2019 (Table 3). Although the initial inorganic nitrogen under RT-RT-RT was the lowest one in all the treatments, the nitrogen absorbed by the crop was also the lowest one. The apparent nitrogen loss was the highest one with 46.11 kg ha^−1^. This indicated that the rotation tillage modes helped to decrease the apparent nitrogen loss, and increase the nitrogen use efficiency.

### 2.10. The Correlation Analysis between Nitrogen Forms and Wheat Yield during 2017–2019

**Figure 7:**
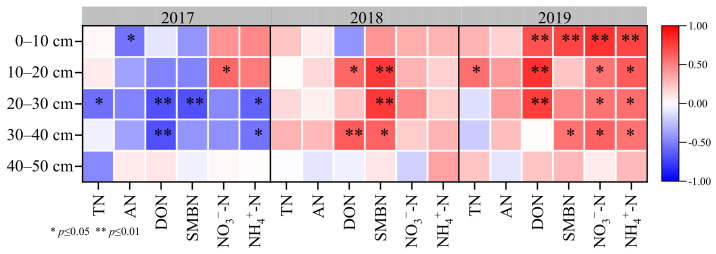
The correlation between nitrogen forms and wheat yield under different soil layers.

The correlation analysis found that the different nitrogen form in different soil layers was negative in the first year of the three-year rotation, while the correlation increased with time (Figure 7). In 2019, except TN and AN, the other nitrogen forms content was all significantly positively correlated with wheat yield in the 0–10 cm soil layer. Additionally, the NO_3_^−^-N and NH_4_^+^-N were positively correlated with wheat yield in the 0–40 cm soil layer.

## 3. Discussion

### 3.1. The Effect of Tillage Practice on Total Nitrogen

Soil total nitrogen (TN) is the pool of nitrogen. It is one of the important indicators to assess soil fertility, but the major component of TN is organic nitrogen [32,33], which needs to transform into inorganic nitrogen such as nitrate nitrogen and ammonium nitrogen, to be absorbed by the crop. Soil tillage practices directly change the soil structure to improve the soil microenvironment [34], further mediating the nitrogen transformation process. Although the TN content is not sensitive to agricultural management, the different tillage practices change the TN vertical distribution by the different disturbance degrees of soil [35,36]. The TN content was changed in the 0–40 cm in the first year by different tillage practices, while the effect was decreased in the 0–30 cm during the following two years in this study. In addition, the effect of deep tillage mainly happened in 10–30 cm, and the combination tillage cycle with deep tillage increased TN content compared with RT-RT-RT. This might be because deep tillage helped to mix the surface soil and the deeper soil, the fertilizer, and the nutrient also mixed and provided the source of organic matter, which led to the TN content accumulation in the deeper soil layer [23,37]. The effect of deep tillage on soil nutrients and structure will decline with time [38]. Our results were in accordance with Han et al. [39] and Zhang et al. [23]. While Wang et al. [40] found that subsoiling—no tillage—subsoiling alternately could increase the TN content in the 0–20 cm soil layer, it did not affect the 20–40 cm soil layer. This might be because the soil disturbance by subsoiling was less than that by deep tillage, and the deep tillage takes more source of fertilizer or crop residue from the surface layer into the deeper soil layer [12,41].

### 3.2. The Effect of Tillage on Nitrogen Components

The nitrogen components were more sensitive than soil total nitrogen to tillage practices. Nitrate and ammonium nitrogen are the major inorganic nitrogen form in the soil, and they are also the main nitrogen form absorbed by the crop [42]. Their content is determined by the transformation between organic and inorganic nitrogen forms [5] and is regulated by interactive processes of production and consumption [43]. Soil microbial biomass nitrogen (SMBN) reflects the microorganism community, it is used to assess the nitrogen transformation process. Dissolved organic nitrogen (DON) is part of nitrogen that is relatively easy to transform. Tillage regimes impact the depth distribution of soil organic matter and affect the soil pore architecture which in turn influences soil aeration, and further regulates the nitrification, denitrification, and the relevant microorganic community and structure, finally affecting the NO_3_^−^-N and NH_4_^+^-N, DON, and SMBN content [44,45]. Mondal et al. [46] reported that soil nitrogen status can be improved through no-tillage adoption particularly in the surface soil layer in a global meta-analysis. Minimum tillage, often in combination with other practices, has been promoted to improve soil health through enhanced microbial activity and increased soil organic matter (SOM) in the surface layer [18,19,20]. In contrast, deep tillage or deep subsoiling was conceived to break up the hard pan in farmland, eliminating soil compaction to boost plant root proliferation, penetration, nutrient uptake, and air permeability [38], improving biological health and physical properties of soil [47], facilitating rain infiltration and water retention [48], and hydraulic conductivity [49]. As a result, deep tillage accelerates the nitrogen transformation and distribution in different soil depths, meanwhile allowing the yield of crops to be continuously enhanced. Our study found that deep tillage promoted NO_3_^−^-N and NH_4_^+^-N transportation into the deeper soil layer, especially for NO_3_^−^-N. However, the effect of deep tillage on DON in deeper layers significantly declined with time. Although the deep tillage significantly increased the SMBN compared with RT-RT-RT, there was no significant change in the same soil layers with time. NO_3_^−^-N cannot be fixed by soil colloid particles, and easily leach with soil water. Deep tillage promotes the soil pore and water storage capacity and helps the soil nitrification and NO_3_^−^-N leaching [48,50]. For DON, although it is relatively easy to transform by the microorganism, there is still part of it belongs to organic form, this might the reason for the shorter affected by the deep tillage.

### 3.3. The Effect of Tillage on Wheat Yield and Nitrogen Balance

The tillage practices affect the soil structure and nutrient cycle, further regulating crop growth and yield [23]. Previous studies showed that although the no-till or minimal tillage profited to increase the soil nutrient in the surface soil layer [19,20,46], the effect on crop yield was different. Generally, no-till is considered shallow compaction or soil hardening by farm machinery traffic can lead to soil constraints to crop growth [51]. In contrast, most studies reported that deep tillage can increase crop yield by breaking up the hard pan in arable land and eliminating soil compaction to boost plant root proliferation, nutrient uptake, and air permeability [43]. As a result, deep tillage increases the plant availability of subsoil nutrients, which increases crop yield if nutrients are growth-limiting and allows the yield of crops to be continuously enhanced [43]. A similar result was found in this study, the wheat yield was increased under treatment with deep tillage compared with RT-RT-RT. Meanwhile, the nitrogen partial productivity demonstrated a similar trend with wheat yield. This indicated that deep tillage improved the nutrient absorbed by wheat and promoted the yield component and wheat yield. The correlation analysis also supported that it was a closer relationship between the wheat yield and nitrogen forms with time.

## 4. Materials and Methods

### 4.1. Site Description

The field experiment was carried out in 2016 at Yuanyang, Henan, China (35°19′ N, 113°50′ E). This area is a warm temperate continental monsoon climate. The mean annual air temperature is 14.5 °C, the mean annual precipitation is 615 mm, and the annual sunshine hours are 2324 h. The soil type is sandy fluvo-aquic soil developed from Yellow River alluviation, which is Calcaric Cambisol according to WBR [52]. The initial soil properties before the experiment in the 0–20 cm soil layer were: organic matter content 17.3 g kg^−1^, total nitrogen 1.00 g kg^−1^, alkaline nitrogen 71.33 mg kg^−1^, available phosphorus 21.6 mg kg^−1^, available potassium 108.0 mg kg^−1^, pH 7.2. The field experiment was a winter wheat (*Triticum aestivum* L. Zhengmai 369)—summer maize (*Zea mays* L. Xundan 29) crop rotation.

### 4.2. Experimental Design

The randomized block design with three replicates was carried out. Five treatments with different combinations of tillage modes with three-year cycles were set as (1) continuous rotary tillage (RT-RT-RT); (2) deep tillage–rotary tillage–rotary tillage (DT-RT-RT); (3) deep tillage–rotary tillage–shallow rotary tillage (DT-RT-SRT); (4) deep tillage–shallow rotary tillage–shallow rotary tillage (DT-SRT-SRT); (5) deep tillage–shallow rotary tillage–rotary tillage (DT-SRT-RT). Each plot was 99.2 m^2^. The information tillage practice before winter wheat seeding during 2016–2018 was demonstrated in Table 4.

The tillage practice is detailed as follows. Summer maize straw was incorporated with all tillage practices. For rotary tillage, a rotary tiller was prepared twice with a depth of 13–15 cm. For deep tillage, first moldboard plows with 28–30 cm, then a rotary tiller was prepared twice with 15–18 cm. For shallow rotary tillage, a rotary tiller was prepared twice with 5–8 cm. The winter wheat was seeded by a seeder machine with a rate of 232.5 kg ha^−1^. The basal fertilizer (N-P_2_O_5_-K_2_O = 20-16-16) was applied 750 kg ha^−1^, and then applied 69 kg N ha^−1^ at the regreening stage in the wheat season. The summer maize and fertilizer were seeded simultaneously with maize density as 67,500 plant ha^−1^ and 750 ha^−1^ component fertilizer (N-P_2_O_5_-K_2_O = 28-10-12).

### 4.3. Soil Sample Collection and Measurement

The soil was sampled after the wheat harvest during 2017–2019. The 0–50 cm depth soil with 10 cm intervals was sampled by the mixture of 5–10 cores. The sample was divided into two parts, one part was stored at 4 °C in the refrigerator to determine soil nitrate nitrogen (NO_3_^−^-N), ammonium nitrogen (NH_4_^+^-N), dissolved organic nitrogen (DON), and microbial biomass nitrogen (SMBN). The other part was air-dried and sieved through 0.85 mm and 0.25 mm to determine the soil alkaline nitrogen (AN) and total nitrogen (TN). The AN was measured by Conway method, and TN was determined by the micro-Kjeldahl method [53]. NO_3_^−^-N and NH_4_^+^-N were extracted from 10 g of fresh soil in 50 mL of 2 mol KCl L^−1^ (1:10 soil: solution ratio) before filtering [54]. The NO_3_^−^-N and NH_4_^+^-N concentrations in the extract were determined using an automated colorimeter (automatic chemical analyzer, Easychem Plus, Via Fratta Rotonda Vado Largo, Italy, Europe).

The dissolved organic nitrogen (DON) content was extracted using the method presented by Gigliotti et al. [55]. Briefly, 10 g of fresh soil with water at a soil-to-water ratio of 1:2 was shaken for 30 min h at 250 rev/min and 25 °C. Next, the supernatant was centrifuged for 10 min at 4000 rev/min before passing through a 0.45 μm membrane filter. The filtrate was measured using a TOC analyzer (Leeman, US17192017, Mason, OH, USA).

The soil microbial biomass nitrogen (SMBN) content was estimated using chloroform fumigation extraction according to the method presented by Vance et al. [56]. Briefly, 20 g of fresh soil was fumigated for 24 h at 25 °C with ethanol-free chloroform, the non-fumigated portion was completed simultaneously. Next, the soils were extracted using 60 mL of 0.5 mol K_2_SO_4_ L^−1^, shaken at 200 rev/min for 30 min, and filtered using filter paper (12.5 cm diameter). The organic nitrogen contents in the extracts were determined using a TOC analyzer (Lehman US17192017). In addition, the SMBN content was calculated according to Jenkinson et al. [57]. as follows: microbial biomass nitrogen = *E*_N_/*k*_EN_, where E_N_ is the D-value between organic nitrogen extracted from fumigated soils and non-fumigated soils; *k*_EN_ = 0.45.

### 4.4. Grain Yield, Yield Components, Aboveground Biomass, and Nitrogen Accumulation

Three replicates of wheat samples (each 1 m^2^) were randomly selected from each plot to measure yield components (spike number per hectare, grain number per spike, and 1000-grain weight) and nitrogen accumulation at the maturity stage. After threshing, drying, and weighing, wheat grain yield, and straw were calculated according to the national wheat grain and straw warehousing standard (at a moisture content of about 14%). The plant samples were oven dried (80 °C) over 48 h and weighed. The grain and straw were divided into two parts, and their nitrogen (N) content was analyzed using the micro-Kjeldahl method (Bao, 2000). Total aboveground nitrogen accumulation was calculated as the grain and straw N content, and the relevant biomass.

We used the certified standard reference materials (bush leaves, GBW07602 (GSV-1); soil, GBW07420), purchased from the National Center of Standard Material in China, to check the measurements.

### 4.5. Calculation

The nitrogen absorbed by aboveground biomass was calculated according to Lu et al. [58].

GNA = GB × GNC(1)

SNA = SB × SNC(2)

ANA = GNA + SNA(3)

where GNA was grain nitrogen accumulation (kg·ha^−1^); GB was grain biomass (kg ha^−1^); GNC was grain nitrogen content (kg kg^−1^); SNA was straw nitrogen accumulation (kg ha^−1^); SB was straw biomass (kg ha^−1^); SNC was straw nitrogen content (kg kg^−1^); ANA was aboveground nitrogen accumulation (kg ha^−1^).

The apparent nitrogen loss (ANL) was calculated based on the ANA according to Xue et al. [59].

ANL = NI − NO(4)

NI = IINS + NAR(5)

NO = ANA + RINS(6)

where ANL was apparent nitrogen loss (kg ha^−1^); NI was nitrogen input (kg ha^−1^), it was the sum of IINS and NAR; IINS was initial soil inorganic nitrogen storage (kg ha^−1^) in the 0–20 cm soil layer; NAR was nitrogen application rate in wheat season (kg ha^−1^); NO was nitrogen output (kg ha^−1^), it was the sum of ANA and RINS (kg ha^−1^); ANA was aboveground nitrogen accumulation (kg ha^−1^); RINS was residue inorganic nitrogen storage (kg ha^−1^) in the 0–20 cm soil layer after wheat harvest in 2019.

### 4.6. Statistical Analysis

Microsoft Excel 2020 (Microsoft Corp., Redmond, WA, USA) was used to input and organize the data, using SPSS Software (ver. 20.0; SPSS Inc., Chicago, IL, USA) for statistical analysis. The ANOVA analysis was used to compare the difference in different nitrogen forms, grain yield, yield components, nitrogen partial productivity, and nitrogen balance indexes between different tillage modes. The multiple comparisons by the least significant range method (LSD) were to analyze the effect of tillage mode, soil depth, and tillage time on the different nitrogen forms. Origin Pro (ver. 8.5; OriginLab Corporation, Northampton, MA, USA) was used to create the graph. All statistical analyses were performed at a significance level of *p* ≤ 0.05.

## 5. Conclusions

The findings carried out from the 3-year cycle tillage experiment showed that the rotation tillage with deep tillage in the first year increased the total nitrogen and the major nitrogen forms content compared with RT-RT-RT. Especially they improved the NO_3_^−^-N and NH_4_^+^-N content in 0–40 cm, with the highest value under DT-SRT-RT. The time, tillage, and depth significantly affected the different nitrogen forms, but there was no effect on DON and SMBN by the interaction of time and tillage. Meanwhile, the rotation tillage promoted the spike number and kernels per spike of wheat, further increasing the wheat yield and nitrogen partial productivity, and with a better effect under DT-SRT-RT. The available nitrogen forms such as NO_3_^−^-N, and NH_4_^+^-N were closely positively correlated with wheat yield in 0–40 cm at with time.

## Figures and Tables

**Table 1:** The three-factor analysis with time, tillage, and soil depth on different nitrogen forms.

Source of Variation	d.f.	TN	AN	DON	SMBN	NH_4_^+^-N	NO_3_^−^-N
Time	2	15.2 **	89.73 **	237.79 **	34.77 **	127.87 **	290.33 **
Tillage	4	4.95 **	20.06 **	22.43 **	28.93 **	6.63 **	21.82 **
Depth	4	2340.33 **	6672.79 **	1218 **	11,731.24 **	6196.85 **	13,686.67 **
Time × Tillage	8	2.84 **	4.93 **	1.86 NS	1.08 NS	7.86 **	10.48 **
Time × Depth	8	8.7 **	37.96 **	152.92 **	18.12 **	10.06 **	35.38 **
Tillage × Depth	16	1.76 *	3.06 **	3.33 **	2.18 **	2.94 **	1.92 *
Time × Tillage × Depth	32	1.65 *	1.39 NS	1.85 **	2.15 **	3.01 **	3.01 **

The source of variation: “Time” means the tillage duration (year); “Tillage” means the five tillage modes; “Depth” means the five sample depths. * represented *p* ≤ 0.05; ** represented *p* ≤ 0.01; NS represented *p* > 0.05.

**Table 2:** Wheat yield, yield components, and fertilizer partial productivity.

Year	Treatment	Spike Number (×10^4^ ha^−1^)	Kernels Per Spike (No.)	Thousand Kernel Weight (g)	Yield (kg·ha^−1^)	N Partial Productivity
2017	RT-RT-RT	634.80 ± 29.55 a	29.53 ± 3.11 a	50.53 ± 2.41 a	6717 ± 103 a	30.67 ± 0.47 a
	DT-RT-RT	602.25 ± 26.25 a	28.83 ± 2.50 a	51.21 ± 1.20 a	6383 ± 208 ab	29.15 ± 0.95 ab
	DT-RT-SRT	586.35 ± 22.95 a	23.47 ± 3.06 b	49.68 ± 1.37 a	5967 ± 148 c	27.25 ± 0.68 c
	DT-SRT-SRT	600.75 ± 19.20 a	24.77 ± 2.67 ab	48.39 ± 0.94 a	6163 ± 191 bc	28.14 ± 0.87 bc
	DT-SRT-RT	598.50 ± 23.70 a	26.17 ± 2.04 ab	49.21 ± 1.60 a	6252 ± 277 bc	28.55 ± 1.27 bc
2018	RT-RT-RT	652.50 ± 31.50 c	25.33 ± 1.53 c	45.5 ± 0.68 a	6096 ± 148 c	27.84 ± 0.68 c
	DT-RT-RT	701.55 ± 20.55 ab	29.00 ± 1.73 ab	46.67 ± 2.04 a	6465 ± 102 ab	29.52 ± 0.47 ab
	DT-RT-SRT	721.35 ± 37.80 a	30.67 ± 2.08 a	45.97 ± 1.24 a	6507 ± 111 a	29.71 ± 0.51 a
	DT-SRT-SRT	677.55 ± 18.00 bc	26.00 ± 2.00 bc	47.72 ± 2.12 a	6259 ± 97 bc	28.58 ± 0.44 bc
	DT-SRT-RT	668.10 ± 16.35 bc	26.83 ± 1.04 bc	46.43 ± 1.50 a	6346 ± 79 ab	28.98 ± 0.36 ab
2019	RT-RT-RT	574.05 ± 12.75 d	28.43 ± 0.98 b	41.89 ± 1.028 c	5719 ± 153 d	26.12 ± 0.7 d
	DT-RT-RT	636.00 ± 8.85 b	28.93 ± 1.00 ab	43.36 ± 1.90 bc	6300 ± 53 b	28.77 ± 0.24 b
	DT-RT-SRT	604.35 ± 6.15 c	26.20 ± 1.80 c	48.57 ± 1.54 a	6003 ± 95 c	27.41 ± 0.44 c
	DT-SRT-SRT	648.30 ± 12.30 ab	30.80 ± 1.00 a	44.13 ± 1.20 bc	6477 ± 36 ab	29.58 ± 0.16 ab
	DT-SRT-RT	667.05 ± 8.10 a	31.00 ± 0.59 a	46.22 ± 0.95 ab	6557 ± 67 a	29.94 ± 0.31 a

Note: Different lowercase letters after the numbers indicate significant differences between different treatments (*p* ≤ 0.05).

**Table 3:** The nitrogen balance under different treatments in 2019.

Treatment	Mineral Nitrogen (kg ha^−1^)	Initial Inorganic Nitrogen (kg ha^−1^)	Nitrogen Absorbed by the Crop (kg ha^−1^)	Residue Inorganic Nitrogen (kg ha^−1^)	Apparent Nitrogen Loss (kg ha^−1^)
RT-RT-RT	219	81.18 ± 0.79 c	173.39 ± 9.73 c	80.93 ± 1.40 c	46.11 ± 2.22 a
DT-RT-RT	219	83.23 ± 0.24 a	197.59 ± 4.40 ab	86.05 ± 0.89 a	18.62 ± 2.54 cd
DT-RT-SRT	219	82.83 ± 1.23 a	190.93 ± 10.26 b	85.55 ± 0.90 a	25.46 ± 2.95 b
DT-SRT-SRT	219	86.70 ± 0.80 bc	200.8 ± 5.02 ab	83.02 ± 0.87 bc	21.89 ± 1.97 bc
DT-SRT-RT	219	84.55 ± 0.73 ab	203.59 ± 3.22 a	84.03 ± 1.64 ab	15.93 ± 3.62 d

Note: Different lowercase letters after the numbers indicate significant differences between different treatments (*p* ≤ 0.05).

**Table 4:** The soil tillage practice before winter wheat seeding during 2016–2018.

Treatment	10/2016	10/2017	10/2018
RT-RT-RT	rotary tillage	rotary tillage	rotary tillage
DR-RT-RT	deep tillage	rotary tillage	rotary tillage
DT-RT-SRT	deep tillage	rotary tillage	shallow rotary tillage
DT-SRT-SRT	deep tillage	shallow rotary tillage	shallow rotary tillage
DT-SRT-RT	deep tillage	shallow rotary tillage	rotary tillage

## Data Availability

Not applicable.
